# HGF/Met Signaling Is a Key Player in Malignant Mesothelioma Carcinogenesis

**DOI:** 10.3390/biomedicines2040327

**Published:** 2014-11-14

**Authors:** Giovanni Gaudino, Haining Yang, Michele Carbone

**Affiliations:** University of Hawai’i Cancer Center, 701 Ilalo Street, Honolulu, HI 96813, USA; E-Mails: hyang@cc.hawaii.edu (H.Y.); mcarbone@cc.hawaii.edu (M.C.)

**Keywords:** malignant mesothelioma, asbestos, erionite, HGF, Met, Akt, SV40, epithelial-mesenchymal transition (EMT)

## Abstract

Malignant mesothelioma (MM) is a highly aggressive cancer related to asbestos or erionite exposure and resistant to current therapies. Hepatocyte Growth Factor (HGF) and its tyrosine kinase receptor Met regulate cell growth, survival, motility/migration, and invasion. HGF and Met are expressed in MM cells, suggesting that the HGF/Met signaling plays a role in development and progression of this tumor, by autocrine and/or paracrine mechanisms. Upregulation and ligand-independent activation of Met, which is under suppressive control of miR-34 family members, correlate with enhanced invasion, migration and metastatic potential in several cancers, including MM. Moreover, Simian Virus 40 (SV40) Tag expression also induces a HGF autocrine circuit in an Rb-dependent manner in human mesothelial cells (HM) and possibly other cell types, enhancing cell adhesion, invasion and angiogenesis. The resulting activation of Met causes HM transformation and cell cycle progression, and contributes to virus particle assembling and infection of adjacent cells. The constitutive activation of Met, frequently occurring in MM, has been successfully targeted in preclinical models of MM. In conclusion, Met expression, activation state, subcellular localization and also HGF co-receptors expression, such as CD44, have clinical relevance for novel targeted therapies in a cancer for which no effective treatment is currently available.

## 1. Introduction

Malignant mesothelioma (MM) is a highly aggressive cancer, resistant to conventional therapies. The vast majority of patients diagnosed with MM die within two years [1]. MM develops from the transformation of human mesothelial cells (HM) of the pleural, pericardial and peritoneal tissues lining body cavities, while about 70% of MM is of pleural origin [2]. The incidence of MM is increasing in the Western world; in the United States approximately 3200 individuals are diagnosed annually with MM (almost none had MM until 1950) and about 100,000 new cases are expected to occur over the next 40 years. A major risk factor for MM is the exposure to asbestos or erionite and many other mineral fibers [2,3]. This cancer is characterized by a long latency (20–50 years) between the first mineral fiber exposure and diagnosis [1]. SV40 (Simian Virus 40) infection and radiation exposure are potential cofactors of MM [4]. Notably, SV40 is the only agent inducing malignant transformation of HM in tissue culture, while asbestos itself is not able to induce HM transformation. However, SV40 infected HM are uniquely susceptible to transformation when exposed to asbestos, with a significant increase in focus formation rate, indicating that SV40 and asbestos are co-carcinogens [5,6].

The fact that only about 5% of individuals exposed to asbestos developed MM and the presence of families with clusters of MM cases suggested that gene-environment interactions were involved in mineral fiber carcinogenesis [2]. We and others identified germline mutations in the BRCA-associated protein 1 (*BAP1*) gene, predisposing for a novel cancer syndrome linked to an inherited very high risk of developing mesothelioma, uveal melanoma, and possibly additional cancers [7,8,9]. BAP1 is a nuclear deubiquitinase, which belongs to the ubiquitin *C*-terminal hydrolase (UCH) family. Although we still ignore the full set of BAP1 targets, its function is likely involved in regulating the activities of Polycomb Group (PcG) and Host Cell Factor-1 (HCF1) target genes, with a central role in regulating gene expression in mammalian cells [10].

A large body of literature established that hepatocyte growth factor (HGF) and its receptor tyrosine kinase Met have a significant role in tumor growth and metastasis and in therapeutic resistance as well. The structural and functional characteristics of the receptor, including its complex biosynthesis, the heterodimeric structure, the signaling mediated by the unique multifunctional docking site, the negative regulation of its activity, make Met stand out from the numerous other receptor tyrosine kinases (RTKs). Meanwhile, its cognate ligand HGF has been characterized as a pleiotropic multifunctional factor, involved both in development and tissue repair, as well as pathological processes such as cancer and metastasis. HGF has a unique dimeric structure, made of an α chain containing four kringle domains and a β chain, linked by a disulfide bond. HGF has been first characterized as a potent mitogen for mature parenchymal hepatocytes and then as a growth factor for a broad spectrum of tissues and cell types. The combination of these characteristics explains why HGF/Met receptor signaling is frequently associated with malignant invasive growth [11,12].

The transforming role of the Met tyrosine kinase was first identified in the fusion protein Tpr–Met oncogene, experimentally induced in human osteosarcoma cells exposed to the mutagen *N*-methyl-*N*'-nitro-*N*-nitrosoguanidine (MNNG), causing translocation of part of Met gene on chromosome seven with the translocated promoter region (TPR) on chromosome one [13]. In the following years, the Met full-length receptor with oncogenic properties was isolated [14] and its structure and biosynthesis were completely described [15,16]. Then, the Met ligand was identified as HGF or Scatter Factor [17,18,19], and other receptors with strong homology with Met, like Sea [20] and Ron [21], were isolated, demonstrating that all members of the Met subfamily share motogenic, mitogenic, and morphogenic activities [22]. The discovery that an exclusive multifunctional docking site, made of a bidentate motif in the receptor *C*-terminal region channeled the signal transduction, revealed the unique high-affinity interactions of activated Met with multiple downstream SH2-containing effectors and prompted the development of novel therapeutic tools for a wide range of malignancies [23].

The signaling of the HGF/Met pair and of its homologs (the Ron ligand is a macrophage-stimulating protein or MSP and is a homolog of HGF [21,24]) are crucial for some steps of embryonic development and tissue regeneration in physiological conditions, while the same signal transduction, when dysregulated, may run the processes of tumor growth and metastasis [11]. Met and HGF-null mice display defects in epithelial-mesenchymal transition (EMT) during organogenesis [25,26]. The elegant *in vivo* approach of disrupting the consensus for Grb2 binding in Met allowed embryos to complete the development with no placental and liver defects, but caused a striking reduction in limb muscle coupled to a generalized deficit of secondary fibers. This important evidence indicates that Met signaling varies in the different tissues during development [27]. Dysregulated MET signaling and consequent aberrant function in human tumors can occur via: (i) gene amplification and overexpression of HGF or Met, (ii) mutation of the receptor kinase domain and other regions, or (iii) establishment of an autocrine loop.

## 2. The Pathogenesis of Mesothelioma

About 60%−70% of pleural MM has been associated with previous exposure to asbestos fibers. The term asbestos refers to six types of natural fibrous silicates characterized by a large-scale use during the 20th century in Western industrial settings, before it was almost completely banned in the 1990s. However, asbestos is still used in some developing countries, because of its combination of good material properties and attractive price. The minerals classified as asbestos are grouped into two major families: serpentine and amphibole, further classified for their chemical composition and crystalline structure. The main member of the serpentine family is chrysotile (also known as “white asbestos”), while amphiboles includes crocidolite (“blue asbestos”), amosite (“brown asbestos”), anthophyllite, actinolite, and tremolite [3]. The biopersistence upon inhalation of chrysotile is relatively low and fibers are quite rapidly cleared from the lungs, while amphibole fibers persist longer in the tissues with fiber concentration proportional to cumulative exposure [28]. It is well accepted that amphibole asbestos fibers cause MM; however, we recently demonstrated that continuous exposure to chrysotile can transform HM *in vitro*, even if the transforming potential is limited when compared with crocidolite amphibole. This is in accordance with the findings of a similar early transcriptional profile upon cell exposure to both mineral types, that remains persistent over time only in cells exposed to crocidolite fibers [29]. Also erionite, a naturally occurring zeolite not contained in the asbestos classification, is a fibrous mineral with *in vitro* transforming potential similar to that of amosite amphibole, albeit with reduced cytotoxicity [30]. Erionite has a powerful carcinogenic potential *in vivo*, causing both pleural and peritoneal MM with higher potency compared to asbestos [2,31]. However, the percentage of MM associated with erionite is smaller, because the exposure to this fiber is usually by far less frequent than that to asbestos [2].

It has been reported that asbestos can induce DNA damage by causing chromosome aberrations affecting segregation during mitosis. Consistently, the resulting structural genomic rearrangements caused by asbestos often involve chromosome types and regions frequently affected in MM [32]. Another known insult of asbestos is genotoxicity, generated by the release of mutagenic reactive oxygen (ROS) and nitrogen (iNOS) species by HM and surrounding macrophages, upon exposure to asbestos fibers [33]. However, cytotoxicity is the prevalent effect of asbestos and it has previously shown that *in vitro* exposure to asbestos can induce apoptosis in a percentage of about 8%−18% of HM population [34,35]. This mechanism eliminates HM with accumulated asbestos-induced mutations, without promoting inflammation, and may be regarded as a strategy to protect against the development of MM [2].

Thus, asbestos cannot transform HM *per se*; on the contrary, the marked cytotoxicity provoked by the fibers tends to cause cell death. The mechanism of asbestos-induced MM carcinogenesis has been elucidated by our discovery that asbestos causes programmed necrosis in the majority of the HM population exposed to the fibers [36]. Necrosis results in the passive release of HMGB1, a damage-associated molecular pattern molecule (DAMP), into the extracellular space [37]. HMGB1 signals the demise of HM by binding with high affinity to RAGE (Receptor for Advanced Glycation End products) of neighboring cells, including macrophages and surviving HM. This induces the secretion of TNF-α and of other cytokines that recruit macrophages and initiate the inflammatory response. Additionally, HMGB1 may activate the Nalp3 inflammasome with consequent secretion of interleukin-1b (IL-1b), also induced by ROS released upon asbestos exposure [38], suggesting a cooperative effect in driving cells toward inflammation. The HMGB1-induced TNF-α release activates Nuclear Factor κB (NF-κB) downstream signaling that generates a survival signal in HM after asbestos exposure. This allows cells with accumulated asbestos-induced DNA damage to survive and propagate the acquired genetic aberrations into the following cell generations, thus becoming prone to transform into malignant clones [39].

The long bio-persistence of asbestos fibers (up to 40−50 years) triggers a malicious cycle of chronic cell death and inflammation that eventually can lead to MM [37]. Interestingly, HMGB1 can also be released by MM tumor cells that become “addicted” to HMGB1 for the maintenance of the transformed phenotype (*i.e.*, tumor cell invasiveness and motility, anchorage-independent growth, *etc.*). Consistently, the pharmacological inhibition of HMGB1 function *in vivo* is suggestive of therapeutic efficacy [40].

**Figure 1:**
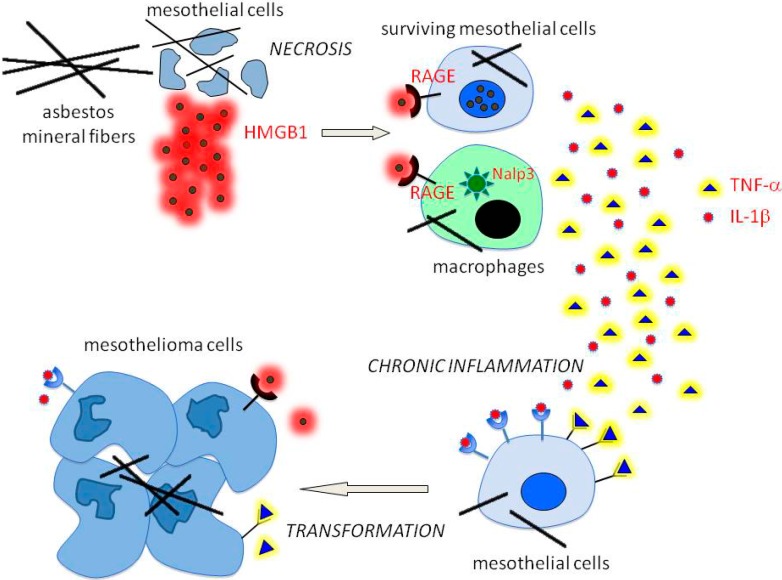
Asbestos and other mineral fibers cause necrotic death of mesothelial cells with the consequent release of HMGB1 in the microenvironment. HMGB1 binds to its receptor RAGE (Receptor for Advanced Glycation End products) of surviving mesothelial cells and macrophages, where it induces Nalp3 inflammasome. As a result, mesothelial cells and macrophages release pro-inflammatory cytokines including TNF-α and IL-1β that cause chronic inflammation, followed by malignant transformation in presence of asbestos-induced DNA damage, and signaling dysregulation.

As a consequence of HM *in vitro* exposure to asbestos, other signal transduction pathways have been found activated. In particular, upon exposure of rat mesothelial cells to crocidolite, the most intensively investigated fiber for its effects on mammalian cells, autophosphorylation of epidermal growth factor receptor (EGFR) and activation of extracellular-regulated kinases 1 and 2 (Erk1/2), with consequent AP-1 transcriptional activity have been reported [41,42]. These pathways are frequently activated in many other cancer types in relation to tumor development and progression (Figure 1).

## 3. HGF/Met Signaling in MM and Potential for Therapy

**Figure 2:**
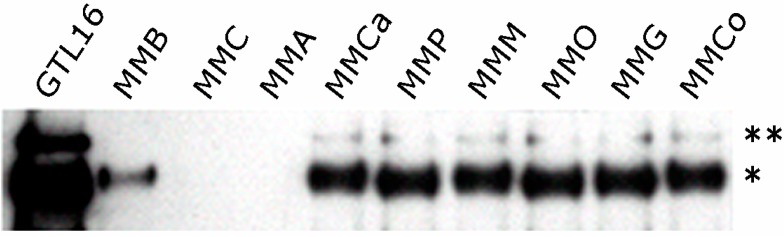
Expression of Met in different MM (Malignant mesothelioma) cell lines. Immunoprecipitation with Met antibodies, followed by immunoblotting with the same antibodies. Asterisks on the right indicate the Met precursor (pr170^MET^, **) and the mature β-chain (p145^MET^, *). GTL16 are gastric carcinoma cells bearing Met amplification and overexpression (control). Modified from [43].

HGF and its tyrosine kinase receptor Met are highly expressed in most MM cells (Figure 2) and tissues [43,44,45].

Moreover, Met expression was found in cells obtained from pleural fluids of patients with mesothelioma, while HGF resulted mitogenic for mesothelial cells. Co-expression of HGF and its receptor was also observed in mesothelioma specimens, indicating a role for HGF/Met signaling in the development of this tumor, either by autocrine or paracrine mechanisms [46]. *In vitro* HGF behaved as a strong chemoattractant for human MM cells and stimulated migration in MM cultured cells that was antagonized by neutralizing HGF monoclonal antibodies. In mesothelioma cells cultured on collagen type IV, HGF induced morphological changes with protrusion of prominent pseudopodia and acquisition of bipolar shape [46]. Secretion of HGF by MM cells was correlated with fibroblast-like or mixed morphology and the functions of secreted HGF were determined according to the cell phenotype: motility and proliferation in epithelioid cells, motility only on fibroblast-like cells [47]. HGF/Met signaling also enhanced MM cell adhesion and invasion, accompanied by expression of matrix metalloproteinases and serine proteases critical for tumor progression [48]. The increased expression of Met in vascular endothelial cells of MM microenvironment and the higher microvessel density in MM overexpressing HGF indicated that a HGF/Met autocrine loop contributed to tumor angiogenesis in MM [49]. Finally, the expression of urokinase-type plasminogen activator receptor (uPAR), the pro-HGF convertase, was induced by exposure of HM to asbestos fibers, suggesting that uPAR-dependent HGF activation is part of the process of pleural mesothelium remodeling [50].

HGF/Met signaling has been reported to be mediated by the phosphatidylinositol-3-kinase (PI3K)/MEK5/Fos-related antigen 1 (Fra-1) pathway [42,51], while that Fra-1 controls the expression of Met and of its co-receptor CD44 [52]. CD44 is a cell surface glycoprotein belonging to the chondroitin sulphate proteoglycan family (CSPG8) and acts as a necessary co-receptor for Met activation and signaling in several cancers and primary cells [53]. Moreover, HGF/Met complex internalization requires CD44, that binds to proteins of the Ezrin-Radixin-Moesin (ERM) family, prompting endosome-originated Met intracellular signaling [54]. Most MMs express CD44, which possibly exerts a relevant function both in the regulation and compartmentalization of Met signaling.

One of the major signals downstream of the activated Met receptor is the phosphatidyl-inositol 3 kinase (PI3K)/Akt pathway. Its signaling reaches the nucleus to affect gene expression and cell cycle progression. Several cellular functions are controlled by Akt, including cell proliferation and survival, cell size and response to nutrient availability, intermediary metabolism, angiogenesis, and tissue invasion. All these processes are hallmarks of cancer and a large body of evidence indicates that dysregulation of Akt activity plays an important role in human cancer. Numerous Akt downstream substrates and effectors have been implicated in tumorigenesis, including mammalian target of rapamycin (mTOR) [55,56]. Receptor-mediated activation of Akt triggers antiapoptotic mechanisms, induces NF-κB transcription, increases tumor invasion and neoangiogenesis, and enhances telomerase activity. It also mediates mRNA translation through mTOR kinase activation, leading to phosphorylation of eukaryotic initiation factor 4E (eIF4E)-binding protein-1 (4E-BP1), which unleashes protein synthesis. mTOR also regulates the activity of the ribosomal protein S6 kinase (p70S6K), controlling cell proliferation and cell cycle progression. Akt pathway is frequently activated in both human and mouse MM [57]. In MM multicellular spheroids and MM tumor fragment spheroids, the role of mTOR in mediating survival signals has been assessed in two *in vitro* clinically relevant three-dimensional settings, suggesting that inhibition of mTOR may provide a valid nontoxic therapy for MM [58,59].

An indirect mechanism of Akt activation in MM occurs through Notch family proteins. MM cells are dependent on Notch-1 signaling as the result of its negative transcriptional regulation on phosphatase and tensin homologue (PTEN), which is the main negative regulator of Akt. Whereas Notch-1 expression was elevated in MM, Notch-2 (which is toxic for MM cells) was down-regulated. The mechanism of Notch-2 toxicity to MM cells antagonized Notch-1 activity, as a result of transcriptional activation of PTEN and consequent inhibition of Akt/mTOR signaling pathway [60].

In MM, anti-apoptotic signaling by HGF/Met is conveyed via Akt and MAPK pathways [61]. Moreover, we have shown that Akt signaling is responsible for resistance to cell death in HM and MM cells after amosite asbestos exposure. Upon exposure to asbestos and other toxic agents, Akt is activated in response to HGF or other growth factors and confers progressive resistance to apoptosis as well as abrogation of CD95/Fas up-regulation [6].

The importance of Met in the process of HM transformation to MM and acquisition of the invasive phenotype was also confirmed by the role played by two microRNA-34 (miRNA-34) family members. The miR-34b and miR-34c were found frequently downregulated by aberrant methylation in MM, resulting in the loss of tumor-suppressive p53 function and the acquisition of a malignant phenotype [62]. However, the same miRNAs have been identified as specific negative regulators of Met expression. The inhibition of these endogenous miRNAs by using antagomiRs dramatically increased Met expression and, conversely, transfection of miR-34b and miR-34c impaired Met signaling and the invasive growth program in cells of lung carcinoma and other cancers [63]. Accordingly, after transfection of miR-34s inhibitors in LP-9 immortalized mesothelial cells and in HM, these miRNAs were downregulated, while cell proliferation, invasiveness, Met expression and Met phosphorylation/activation were significantly increased with the onset of the oncogenic phenotype. This suggests a key role of the balance between miR-34 family members and Met in the early carcinogenic process of MM [64].

The function of Met can be dysregulated by mutation, amplification, overexpression or autocrine activation, with consequent acquisition of the oncogenic phenotype by cells harboring the altered receptor. This suggests that Met may be a valid therapeutic target, by using tools able to interfere with its kinase activity or with the HGF ligand interaction. To date, several drugs targeting Met and HGF have been investigated *in vitro* and *in vivo* [65].

The first generation small molecule Met inhibitor SU11274 has been successfully used to impair proliferation, wound healing and motility of H28 MM cells, by inhibiting Met kinase activity and downstream phosphorylation of Erk1/2 and Akt [44]. The second generation small molecule Met inhibitor, PHA665752, has been shown effective in MM, blocking the phosphorylation of Met, Akt, Erk1/2 and p70-S6K, *in vitro* alone [45] or in combination with rapamycin, revealing that combination targeting of mTOR and Met suppresses Akt pathway activation and more effectively decreases cell growth [66]. Moreover, frequent co-expression of Met and EGFR in MM cell lines and tumors generates a cross-talk of the relative pathways. Knockdown of Met by RNA interference inhibited not only the phosphorylation of Met but also that of EGFR. Conversely, the stimulation with HGF increased both Met and EGFR tyrosine phosphorylation. The combination of PHA-665752 Met inhibitor and the EGFR inhibitor, erlotinib, suppressed MM cell growth with an additive effect, as occurred by combinations of rapamycin with different RTK inhibitors. These results revealed that combination targeting of kinase signaling pathways is more effective than single agents in most MM. Interestingly, the effect is particularly striking, especially when the receptor activation occurs through a HGF/Met autocrine loop [67].

The NK4 protein is a fragment of HGF, consisting of an *N*-terminal hairpin domain and 4 kringle domains of the α-chain of HGF, generated by peptidase HGF digestion in mast cells and neutrophil during inflammation [68]. Recombinant NK4 has been characterized as a competitive inhibitor of HGF for Met binding and it has been proposed as a potential targeting drug in case of tumorigenic processes driven by HGF/Met paracrine or autocrine mechanisms [69]. In MM cultured cells, NK4 inhibited Met activation and suppressed cell proliferation, migration and invasiveness. In a subcutaneous xenograft model of MM, adenovirus-mediated intratumoral expression of NK4 significantly inhibited tumor growth, also due to a strong inhibition of tumor neoangiogenesis due to the known anti-angiogenic potential of NK4 [70].

Antibodies directed against a v6-encoded epitope of CD44 or CSPG8, the co-receptor of Met, inhibited receptor activation and signaling, and abrogated tumor growth and metastasis in a fibrosarcoma model [71]. In MM we showed that chondroitin sulphate proteoglycan 4 (CSPG4), belonging to the same family of CD44, is expressed in MM cell and biopsies, and monoclonal antibodies against CSPG4 significantly reduced MM cell motility, migration, invasiveness, and anchorage-independent growth. CSPG4 antibodies also prevented or inhibited MM xenografts in SCID mice with a significant increase in animal survival. CSPG4 also inhibited Akt activity, with pro-apoptotic effects on MM cells [72]. At present there is no evidence that CSPG4 shares with the homolog CD44 a cooperative function with Met receptor activity. However, the common pathways affected and the converging effects obtained with neutralizing antibodies suggest an intriguing role for anti-CSPG4 in antagonizing Met mediated carcinogenesis.

## 4. SV40 Replication and Met in Mesothelial Cell Transformation

Simian virus 40 (SV40) is a DNA virus isolated in 1960 from contaminated polio vaccines, that induces mesotheliomas, lymphomas, sarcomas, brain and bone tumors in hamsters. In humans, the same tumor types have been shown to contain SV40 DNA and proteins. MMs and brain tumors are most consistently associated with SV40, with a range of positivity from 6% to 60% [73].

*In vitro* and animal experiments show co-carcinogenicity between SV40 and asbestos in HM that are particularly susceptible to SV40 transformation [5,6], possibly because in SV40-transformed HM abundant viral DNA persists in episomal form without completing the viral cycle. This is possible because of a mechanism suppressing viral late gene expression by antisense RNAs, as a result of extension of the early transcripts beyond the early termination signal into the late region [74]. As a consequence, the early gene product SV40 large T antigen (Tag) accumulates in infected HM representing the main SV40 oncogene.

Tag interacts with multiple intracellular signaling pathways and in particular forms a Tag-p53 complex that recruits retinoblastoma protein, p300, p400 and CREB-binding protein, leading to a powerful transcriptional complex able to activate IGF-1, Met and Notch-1 expression and the relative pathways [75].

**Figure 3:**
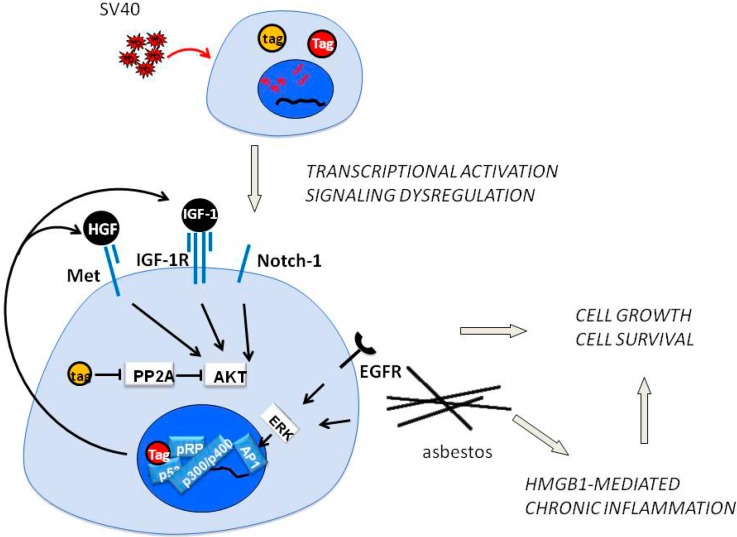
Upon SV40 infection of mesothelial cells, Tag (Large T) and tag (small t) antigens are expressed. Tag binds p53, pRb, p300/400, forming a transcriptional complex that induces expression of HGF (Hepatocyte Growth Factor), IGF-1, other growth factors and the Notch-1 receptor. The consequent autocrine circuits converge on Akt, Erk and other pathways, leading to cell growth and survival. The inhibitory effect of tag on PP2A phosphatase contributes to Akt activation. The exposure to asbestos activates EGFR and the Erk/AP1 axis, and induced programmed cell necrosis, followed by the HMGB1/TNF-α/NF-κB pathway and chronic inflammation.

We also showed that Tag expression induces a HGF autocrine circuit in Rb-dependent manner in HM, and possibly in other cell types. The resulting activation of HGF receptor Met causes EMT of HM and S-phase entry, cell cycle progression, virus particle assembling and infection of adjacent cells. This mechanism may explain how a limited number of SV40-positive cells may be sufficient to direct non infected HM toward malignant transformation [43]. Moreover, we also demonstrated that SV40 infection of HM and astrocytes induced an increase of Met and Notch-1 expression, and this effect was specific because the expression of other tyrosine kinase receptors in HM and astrocytes (e.g., EGFR, PDGFR) were not influenced by SV40 infection. We found that the downstream effector of Met, Akt-1 was phosphorylated/activated in SV40-infected cells. Akt protein levels were not affected and PTEN expression, the negative regulator of the PI3K/Akt/mTOR axis, was not influenced by SV40 infection suggesting that, in SV40-infected human cells, Met was the main regulator of Akt [76] (Figure 3).

In this model, a sustained level of Tag expression is critical for transformation, while host cell target proteins also play an important role. SV40 activates a HGF/Met autocrine loop, driving accelerated and invasive cell growth [43], while Notch-1 is transcriptionally induced by SV40 early proteins in infected HM [77]. However, Met activation induces Notch function, which in turn down-regulates Met at transcriptional level, suppressing HGF-dependent invasive growth program [78]. In HM and astrocytes, SV40 activates Met and Notch-1, possibly altering the Met-Notch negative feedback, thus facilitating cellular transformation [76].

## 5. Epithelial-Mesenchymal Transition, Met and Mesothelioma

Epithelial-mesenchymal transition (EMT) is a physiological process occurring during development, when progenitor cells need to migrate over long distances in the embryo. One of the more powerful regulators of EMT is HGF/Met signaling, as in the case of EMT during myogenesis [27,79]. However, EMT is also the process allowing epithelial and mesothelial cells to acquire a mesenchymal phenotype and properties associated with cell migration, invasiveness and cancer progression, as in the case of colon cancer cells and other cancers driven by dysregulated Met signaling [80]. EMT occurs during MM oncogenesis and it has been reported as a critical process also, for determining the morphological features of this cancer, with a relevant impact on prognosis. The histopathological subtypes were correlated with a more favorable prognosis in epithelioid mesothelioma, worse in tumor of the biphasic type, and worst in sarcomatoid MMs [81,82,83]. Also microRNA (miRNA) expression profiles have been associated with the histopathological subtypes [84], suggesting diagnostic and prognostic significance [85]. Notably, miRNAs have been implicated in the regulation of EMT [86]. Finally, a novel classification of MM based on different molecular profiles, gene alterations (including Bap1 mutated status) and survival outcomes, confirmed at the molecular level that dysregulated EMT is an important parameter to differentiate the two subgroups identified [87].

As well as Met, the two main players in MM oncogenesis are also strong mediators of EMT. HMGB1 has been associated with EMT in alveolar epithelial cells [88,89] and TNF-α has been shown to induce EMT in mesothelial cells [90]. Moreover, recent evidence in clear cell renal cell carcinoma (ccRCC) indicates that HMGB1/TNF-α and HGF/Met/Akt pathways may converge toward EMT regulation. In ccRCC cells TNF-α was shown to induce EMT and the expression of CD44, the Met c o-receptor, leading to poor prognosis, invasiveness, metastasis and resistance to targeted sunitinib therapy [91]. At present, the common link between MM and ccRCC is limited to the fact that both cancers share a relevant percentage of Bap1 mutational loss [7,92]. However, it is possible that a cross-talk between HMGB1/TNF-α and HGF/Met/Akt may occur in MM also, supporting and reinforcing the process of MM carcinogenesis and tumor progression.

**Figure 4:**
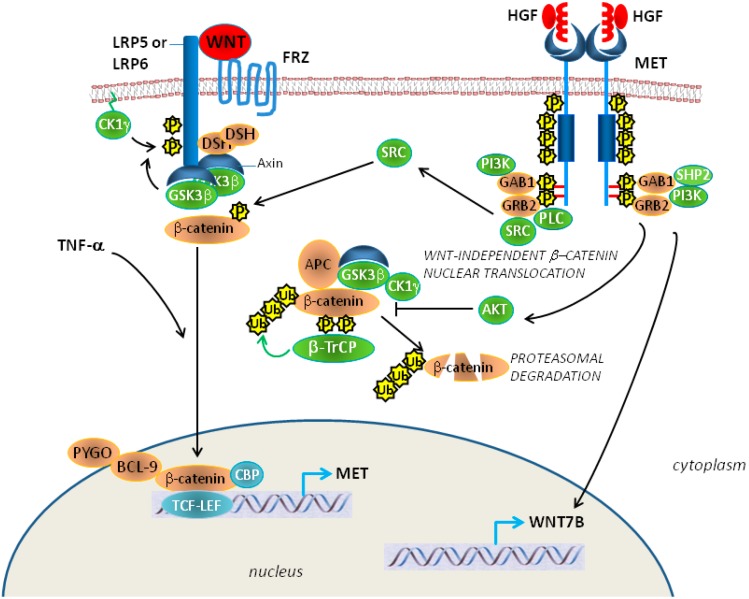
Met and Wnt-β-catenin signaling pathways cooperate in regulating EMT (epithelial-mesenchymal transition). MET contributes to transcriptional activation of Wnt ligands, such as WNT7B. Met contributes to nuclear translocation of β-catenin by its tyrosine phosphorylation (directly or indirectly by SRC), or by inhibition of the β‑catenin degradation complex by Akt that phosphorylates glycogen synthase kinase‑3β (GSK3β). β‑TrCP, β‑transducin repeat-containing protein; APC, adenomatous polyposis coli; CBP, CREB-binding protein; CK1, casein kinase 1; DSH, disheveled; FRZ, frizzled; GAB1, GRB2‑associated-binding protein 1; GRB2, growth factor receptor-bound protein 2; LRP, low-density lipoprotein receptor-related protein; PLC, phospholipase C; TCF-LEF, β-catenin target genes; TNF-α, Tumor Necrosis Factor-α; PYGO, pygopus. Modified from [80] with permission of NPG.

During the process of EMT the expression of a number of mesenchymal markers such as the cytoskeletal proteins, vimentin, and α-smooth muscle is increased; whereas the epithelial cell adhesion molecule E-cadherin is downregulated, either at transcriptional level or by post-transcriptional ubiquitin-mediated degradation. E-cadherin is part of cell adherent junctions in a multiprotein complex including β-catenin, a key component of the canonical Wnt pathway, which is mainly regulated by serine/threonine phosphorylation. A large body of evidence reported the association of Met with β-catenin (Figure 4), followed by Wnt-independent nuclear translocation in human tumor cells [93,94,95].

A recent report shows that TNF-α pro-invasive activity requires Met signaling to sustain Mek/Erk activation and Snail accumulation, leading to E-cadherin down-regulation. TNF-α induces Met transcription via NF-κB and in human colorectal cancer tissues high levels of TNF-α correlate with increased Met and HGF expression, responsible for HGF/Met pro-invasive paracrine circuits in these tumors [96]. These results further support the existence of a cross-talk between the HMGB1/TNF-α/NF-κB axis and the HGF/Met pathway.

We showed that the majority of established MM cell lines exhibit nuclear accumulation of β-catenin, suggesting a possible contribution of β-catenin in MM development. Indeed, the exposure to either crocidolite or chrysotile fibers resulted in EMT of HM, with consequent E-cadherin downregulation and β-catenin phosphorylation on tyrosine 142 (Y142) by the Met tyrosine kinase receptor (and other RTKs). This eventually resulted in disassembling of adherent junction and release of β-catenin, followed by nuclear translocation [29].

## 6. Conclusions

HGF and its tyrosine kinase receptor Met are key players in MM carcinogenesis and tumor progression. Autocrine and paracrine mechanisms involving this ligand-receptor system lead to the activation of signaling pathways like PI3K/Akt/mTOR axis and MAPK/Fra-1 pathway that are of high relevance in mesothelial transformation upon exposure to carcinogenic mineral fibers. Dysregulation of Met receptor or of HGF ligand promotes cell growth, survival, angiogenesis and metastasis. The expression of Met and the onset of an autocrine loop with HGF contribute to SV40 co-carcinogenicity with asbestos, prompting for preventive and therapeutic intervention. The morphological changes occurring during the mesothelial transformation caused by asbestos and other carcinogenic mineral fibers involve epithelial-mesenchymal transition (EMT), coordinated by Met signaling at cytosolic and nuclear levels. Targeted therapy against HGF, Met and their downstream pathways is promising, either as monotherapy or in combination with other specific drugs. However, at present the body of evidence supporting HGF/Met signaling as a therapeutic target for MM is still limited. Therefore, a solid validation of this hypothesis is needed by further preclinical and clinical studies with the available specific Met inhibitors (small-molecules, antibodies, and siRNAs). The search for novel therapeutic tools for MM based on Met targeting will contribute to the acquisition of effective therapies for this almost invariably fatal disease.
